# Synaptotagmin-7 Is an Asynchronous Calcium Sensor for Synaptic Transmission in Neurons Expressing SNAP-23

**DOI:** 10.1371/journal.pone.0114033

**Published:** 2014-11-25

**Authors:** Jens P. Weber, Trine L. Toft-Bertelsen, Ralf Mohrmann, Ignacio Delgado-Martinez, Jakob B. Sørensen

**Affiliations:** 1 Department of Neuroscience and Pharmacology, Faculty of Health Sciences, University of Copenhagen, Copenhagen, Denmark; 2 Department of Functional Genomics, Center for Neurogenomics and Cognitive Research, Vrije Universiteit, Amsterdam, The Netherlands; 3 Department of Physiology, University of Saarland, Homburg, Germany; 4 Singapore Institute of Neurotechnology, National University of Singapore, Singapore; 5 Lundbeck Foundation Center for Biomembranes in Nanomedicine, University of Copenhagen, Copenhagen, Denmark; University of Waterloo, Canada

## Abstract

Synchronization of neurotransmitter release with the presynaptic action potential is essential for maintaining fidelity of information transfer in the central nervous system. However, synchronous release is frequently accompanied by an asynchronous release component that builds up during repetitive stimulation, and can even play a dominant role in some synapses. Here, we show that substitution of SNAP-23 for SNAP-25 in mouse autaptic glutamatergic hippocampal neurons results in asynchronous release and a higher frequency of spontaneous release events (mEPSCs). Use of neurons from double-knock-out (SNAP-25, synaptotagmin-7) mice in combination with viral transduction showed that SNAP-23-driven release is triggered by endogenous synaptotagmin-7. In the absence of synaptotagmin-7 release became even more asynchronous, and the spontaneous release rate increased even more, indicating that synaptotagmin-7 acts to synchronize release and suppress spontaneous release. However, compared to synaptotagmin-1, synaptotagmin-7 is a both leaky and asynchronous calcium sensor. In the presence of SNAP-25, consequences of the elimination of synaptotagmin-7 were small or absent, indicating that the protein pairs SNAP-25/synaptotagmin-1 and SNAP-23/synaptotagmin-7 might act as mutually exclusive calcium sensors. Expression of fusion proteins between pHluorin (pH-sensitive GFP) and synaptotagmin-1 or -7 showed that vesicles that fuse using the SNAP-23/synaptotagmin-7 combination contained synaptotagmin-1, while synaptotagmin-7 barely displayed activity-dependent trafficking between vesicle and plasma membrane, implying that it acts as a plasma membrane calcium sensor. Overall, these findings support the idea of alternative syt∶SNARE combinations driving release with different kinetics and fidelity.

## Introduction

Synaptic transmission depends on the fusion of synaptic vesicles with the plasma membrane and the ensuing neurotransmitter release [1]. The triggering speed of synaptic vesicle fusion varies widely. In the Calyx of Held, the increase and decay of the release rate takes place within ∼1 ms, resulting in a highly synchronized burst of glutamate release [2]. In contrast, in synapses formed by cholecystokinin-containing GABAergic interneurons, secretion of neurotransmitter persists for >100 ms after a single action potential [3]–[5]. Asynchronous release dominates during and immediately following trains of stimuli in many synapses [6]–[9], but is also measurable following single stimuli [10], [11].

Synaptic vesicle exocytosis depends critically on the ternary SNARE-complex, which forms between vesicle and plasma membrane [12], [13]. At least synaptotagmin-1, and -2 (henceforth referred to as syt-1 and syt-2) act as calcium-sensors for synchronized release in many glutamatergic and GABAergic synapses [14]. The effect of removing syt-1 or -2 is a loss of synchronous release, combined with persisting or augmented asynchronous release and – in most but not all systems – an increase in spontaneous release rate [14].

The identity of the calcium sensor for the asynchronous phases of release has remained unknown until recently. Synaptotagmin-7 (henceforth referred to as syt-7) is highly expressed throughout the central nervous system [15], [16], including in the presynaptic compartment [16], [17]. Syt-7 was first found to constitute the asynchronous calcium sensor for neurotransmitter release at the zebrafish neuromuscular junction [18]. In central synapses, deletion of syt-7 does not affect basal synaptic transmission upon single stimulation [19]. However, knock-down of syt-7 was recently found to strongly decrease asynchronous release in syt-1 knockout neurons and to mildly depress asynchronous release during action potential trains in wildtype neurons [20]. Another investigation also found that syt-7 elimination inhibited release during high-frequency stimulation, but further studies led to the conclusion that syt-7 acts upstream of syt-1, as a calcium-sensor for vesicle replenishment [21].

Syt-7 has previously been shown to be a major vesicular calcium sensor for dense core vesicle exocytosis in endocrine cells [22]–[27] and for lysosome fusion [28]. A moderate delay in neuronal outgrowth from superior cervical ganglion neurons was identified in a syt-7 knock-out mouse [29]. Overexpression studies identified different roles for syt-7 splice variants in synaptic vesicle recycling [30].

An unresolved question is how syt-7 interacts with the SNARE-proteins, which constitute the ‘blue-collar’ workers that execute membrane fusion itself [13]. Investigations of the interaction between SNAP-25 and syt-1 have identified negative charged residues on SNAP-25, which appear to interact directly with syt-1 [31], [32]. These charged amino acid residues are situated around the middle of the four helical SNARE-bundle, facing the outside of the complex, and their mutation both copy and occlude syt-1 deletion in adrenal chromaffin cells [33]. However, slow secretion is still present upon mutation of these residues within the SNAP-25A isoform, and therefore it appears that the alternative calcium sensor in this case (presumably syt-7 [22]) interacts with the SNAREs in a different mode. Alternatively, syt-7 might interact with a different set of SNAREs altogether. Indeed, in previous work using an *in vitro* docking assay the almost ubiquitously expressed SNAP-23 associated specifically with syt-7 expressing granules to cause vesicle docking, whereas docking in the presence of SNAP-25 depended on syt-1 [34].

Here, we studied the molecular basis for a specific form for asynchronous release, which is induced in SNAP-25 knock-out (KO) hippocampal glutamatergic neurons after expression of SNAP-23 [35]. By generating a SNAP-25/syt-7 double knock-out (DKO) mouse we show that SNAP-23 driven asynchronous release depends on syt-7. These data indicate that syt isoforms associate with specific Q-SNAREs to trigger exocytosis with different kinetics.

## Materials and Methods

### Ethics statement

Permission to keep and breed knockout mice for this study was obtained from The Danish Animal Experiments Inspectorate. The animals were maintained in an AAALAC-accredited stable in accordance with institutional guidelines as overseen by the Institutional Animal Care and Use Committee (IACUC). Adult mice were sacrificed by cervical dislocation; embryos were sacrificed by decapitation. In vivo experiments were not carried out.

### Mouse lines and cell culture

SNAP-25 knock-out (−/−) embryos of either sex expressing endogenous syt-7 were obtained by crossing SNAP-25 heterozygotes and performing cesarean section on embryonic day 18 (E18). To create embryos of either sex lacking both SNAP-25 and syt-7, the two knockout mouse lines [19], [22], [36] were crossed and in the second generation animals knock-out for syt-7 (−/−) and heterozygous for Snap25 (+/−) were identified by PCR genotyping. These animals were viable, in agreement with the finding that elimination of syt-7 does not impair survival in mice [19]. Crossings allowed us to isolate SNAP-25/syt-7 double knock-out (DKO) embryos at embryonic day 18 (E18). Astrocyte feeder islands and hippocampal neurons were prepared as described previously [35]. For electrophysiology, isolated hippocampal neurons were plated on astrocyte microislands [37] in Neurobasal medium (Invitrogen, Carlsbad, CA) supplemented with B-27 (Invitrogen), 17.3 mM HEPES, 1% GlutaMax-I (Invitrogen), 1% penicillin/streptomycin (Invitrogen), 25 µM β-mercaptoethanol. Neurons were allowed to mature for 10–14 days before they were used for experiments. Only islands containing single neurons were examined.

### Lentiviral vectors

The lentivirus plasmid encoding SNAP-25B or SNAP-23 fused N-terminal to Enhanced Green Fluorescent Protein (EGFP) has been described before [35]. A 25 amino acid linker separates the EGFP from the complete SNAP-23 or -25 open reading frame. For experiments carried out in combination with syt-pHluorin expression, we used Enhanced Cyan Fluorescent Protein (ECFP) fusion proteins, where the EGFP ORF was replaced with ECFP and remained separated by a 24 amino acid linker from SNAP-25B or SNAP-23. Constructs coding for ecliptic GFP (pHluorin)∶synaptotagmin fusion proteins were generated in analogy to pHluorin-synaptotagmin I reported by Diril et al. [38]. Employing PCR overlap extension with engineered primers, the coding sequence for each paralog/isoform (syt-1, syt-7s, and syt-7L; [16]) was N-terminally fused to the sequence of pHluorin using a short linker that also encoded a Tobacco Etch Virus cleavage site (amino acid sequence: DYDIPTTENLYFQGELKTVDAD, cleavage site underlined). To ensure that the resulting fusion protein is correctly inserted into the membrane despite its N-terminal modification, we further added the signal sequence of preprotachykinin to the N-terminus of the open reading frame (amino acid sequence: MKILVAVAVFFLVSTQLFAEEIGAN). PCR products were then inserted into the multiple cloning site (restriction sites: Xba I/NheI) of the p156rrL lentiviral shuttle vector, which carried the WPRE sequence downstream of the expression cassette. The vector was previously modified to contain the synapsin promotor for neuron-specific expression. All constructs were verified by sequencing. Lentiviruses were produced, aliquoted and frozen as previously described [35]. To investigate the role of syt-7 in synaptic transmission, neurons from SNAP-25 knockouts (expressing endogeneous syt-7) were infected with lentiviruses expressing EGFP-SNAP-25 or EGFP-SNAP-23, and compared with neurons from SNAP-25/syt-7 double-knockouts expressing the same two constructs.

### Immunocytochemistry

Hippocampal neuronal cultures on Poly-Ornithine-Laminin coated coverslips were fixed for 15′ at RT in 3.7% formaldehyde. After three washes in PBS, fixed cells were permeabilized using 0.5% Triton-X-100 for 5 min and afterwards incubated in PBS containing 2% goat serum and 0.1% Triton-X-100 for 60 min to block nonspecific binding. Cultures were incubated for 2 h with primary antibodies in the presence of goat serum (2%) and 0.1% Triton-X-100. The primary antibodies used were anti-Syt-1 (1∶1000, mouse monoclonal, Synaptic Systems, Germany, Cat.No. 105 011), and anti-Syt-7 (1∶1000, rabbit polyclonal, Abcam, USA, Cat.No. 105 173). The cells were washed three times with PBS and then incubated over night at 4°C with secondary antibodies: Alexa 546-coupled goat-anti-mouse (Invitrogen) and Alexa 637-coupled goat-anti-chicken (Invitrogen), both diluted 1∶500. Immunofluorescence images were taken with a confocal microscope (LSM 510 utilizing 488 nm, 543 nm and 633 nm lasers controlled by LSM 5 software attached to an Axiovert 200; Zeiss, Germany) using a 63× oil immersion (1.4 NA) objective. For the 546 nm channel, a HFT 488/543 Dichroic Beam Splitter (BS) and a 560–615 BP Filter were used; HFT 488 BS and 505–530 nm BP Filer for the 488 nm channel and a HFT UV/488/543/633 combined with a 650 nm LP filter for the 633 nm channel (all Zeiss, Germany).

### Electrophysiology

Autaptic cells between DIV 10 to 14 were used for experiments. The patch-pipette solution included 135 mM K-gluconate, 10 mM HEPES, 1 mM EGTA, 4.6 mM MgCl_2_, 4 mM Na-ATP, 15 mM creatine phosphate, and 50 U/ml phosphocreatine kinase, 300 mOsm, pH 7.3. The standard extracellular medium consisted of 140 mM NaCl, 2.4 mM KCl, 10 mM HEPES, 10 mM glucose, 4 mM CaCl_2_, and 4 mM MgCl_2_, 300 mOsm, pH 7.3. Cells were whole-cell voltage clamped at −70 mV with an EPC-9 amplifier (HEKA Elektronik, Lambrecht/Pfalz, Germany) under control of Pulse 8.80 software (HEKA Elektronik). Currents were low-pass filtered at 1 or 5 kHz and stored at either 10 or 20 kHz. The series resistance was compensated 75%. Only cells with series resistances below 20 MΩ were analyzed. Pipette resistance ranged from 4 to 6 MΩ. All recordings were made at room temperature. EPSCs were evoked by depolarizing the cell from −70 to 0 mV for 2 ms. Solutions were exchanged via a fast local multibarrel perfusion system (Warner SF-77B, Warner Instruments, USA). The patch pipettes were made of borosilicate glass and pulled using a multi-step puller (P-87; Sutter Instruments, Novato, CA). Sucrose experiments were performed with 500 mM Sucrose in Tetrodotoxin (TTX, 0.5 µM) containing extracellular solution as described by [39]. Recording of miniature EPSCs (mEPSCs) was performed in the presence of 0.5 µM TTX. Spontaneous events were detected using Mini Analysis program (Synaptosoft, USA).

### Live cell imaging

Hippocampal autaptic cell cultures at DIV 14–21 were used for imaging. Standard extracellular solution identical to the one used in electrophysiological measurements was used. For the acidification test HEPES in the extracellular solution was replaced by MES (2-[N-morpholino]ethanesolfonic acid), and the pH was adjusted to pH 5.5. Ammonium chloride solution was made by substituting 50 mM NaCl with NH_4_Cl (pH was adjusted to 7.40). Stimulation of cells was done by superfusing the cells with an extracellular solution where 45 mM NaCl was substituted with 45 mM KCl to depolarize the membrane. Images of live cells were taken using a Andor 885 EM-CCD connected on a Zeiss Axio Observer M inverted microscope. To separate ECFP and EGFP signals according to their excitation spectra, narrow nm-precise light stimulation using a monochromator (TiLL Polychrome V) was used at 475 and 490 nm, respectively. TILL Live Acquisition software was used for data acquisition and timing was coordinated with a gravity driven local application system placed on a stepper motor (Warner SF-77B) using HEKA Patchmaster Software. A three frame baseline was taken, followed by a 30 s KCl application and a 10 s acid and ammonium wash. Pictures were taken at a 1 s interval during baseline and stimulation, and at the end of the washing steps. SNAP-23/-25 expression was confirmed by ECFP fluorescence.

### Statistics

Results are shown as average +/− SEM, with n referring to the number of cells from each group, unless otherwise noted in the text. n is given on the bar charts or the figure legends. We tested the effect of eliminating syt-7 in the presence of SNAP-25 (the group SNAP-25+syt-7 against the group SNAP-25−syt-7), and – in separate tests – in the presence of SNAP-23 (the group SNAP-23+syt-7 against the group SNAP-23−syt-7). In case of homoscedastic data, the groups were compared using a Student's t-test. For heteroscedastic data, non-parametric Mann-Whitney Test was used. Significance was assumed when p<0.05. Statistical testing was done using Origin Pro 8 (OriginLabs, USA).

## Results

### Syt-7 triggers asynchronous release in the presence of SNAP-23

**Figure 1:**
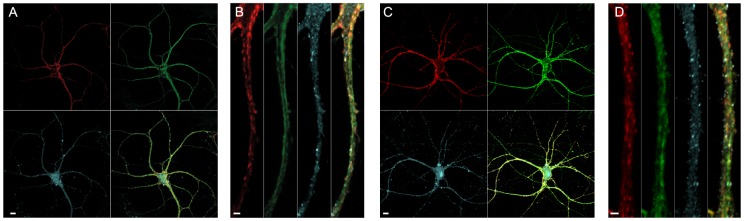
Synaptotagmin-7 staining in cultured hippocampal neurons overlaps with both synaptotagmin-1 and overexpressed SNAP-23/SNAP-25. A: Hippocampal neuron overexpressing EGFP-SNAP25 (green channel, upper right) and stained for syt-1 (red, upper left) and syt-7 (blue, lower left). Scale bar = 10 µm. The lower right panel shows the combined image. B: Close-up image shows that syt-1 is concentrated in spots, which represent presynapses, whereas both EGFP-SNAP25 and syt-7 have a more wide-spread distribution. Scale bar = 2 µm. C: Hippocampal neuron overexpressing EGFP-SNAP23 (green channel, upper right) and stained for syt-1 (red, upper left) and syt-7 (blue, lower left). Scale bar = 10 µm. The combined picture is shown in the lower right panel. D: Close-up image shows a widespread distribution of both EGFP-SNAP-23 and syt-7, which overlaps with, but is not restricted to, the areas stained for syt-1. Scale bar = 2 µm.

Previous experiments showed that expression of SNAP-23 in SNAP-25 knock-out (KO) hippocampal neurons restores neuronal survival, as well as neurite and synapse numbers; however the ensuing evoked release is strongly asynchronous [35]. Here, we expressed N-terminally EGFP-tagged SNAP-25B – which is fully functional [40] – or EGFP-SNAP-23 in cultured hippocampal neurons using a lentiviral expression system [35]. Confocal microscopy showed that both SNAREs distributed to the entire neuritic tree (Fig. 1A–D), as previously reported [35]. Simultaneous co-staining against syt-1 and syt-7 showed that the former is enriched in distinct spots, which presumably represents pre-synapses, whereas syt-7 appears to be more widespread [30], but its distribution overlaps with syt-1, EGFP-SNAP-25B and EGFP-SNAP-23 (Fig. 1B, D).

To investigate whether specific association of SNAP-23 with syt-7 could explain asynchronous release in the presence of SNAP-23, we crossed SNAP-25 heterozygote mice with the previously described mouse line bearing a neomycin cassette in the syt-7 gene [19]. These mice do not express detectable levels of syt-7 and will therefore here be referred to as syt-7 knockouts (KO). Since syt-7 KO mice are viable, we created a mouse line, which was homozygous knock-out for syt-7, and heterozygous for SNAP-25. Crossing of these animals made it possible to recover SNAP-25/syt-7 DKO mice by cesarean section at E18. As a control, we performed parallel experiments using SNAP-25 KO E18 animals.

**Figure 2:**
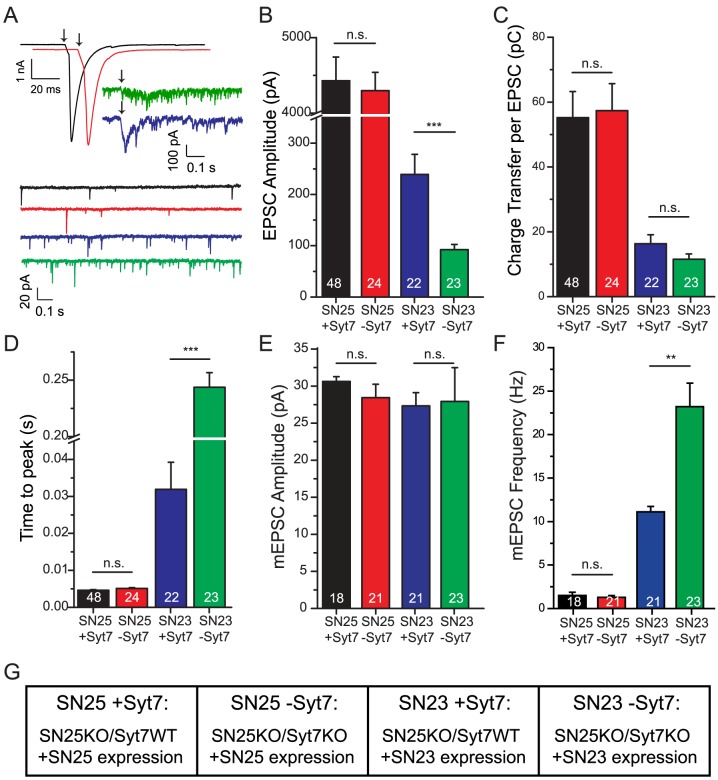
Expression of SNAP-23 in SNAP-25 KO neurons leads to asynchronous release, which is exacerbated by deletion of Synaptotagmin-7. A, top: Representative EPSC traces of SNAP-25 KO neurons rescued with SNAP-25 (black) or SNAP-23 (blue) and SNAP-25/Syt-7 DKO neurons rescued with SNAP-25 (red), or SNAP-23 (green). Stimulation is indicated by an arrow. A, bottom: Representative mEPSC traces of SNAP-25 KO neurons rescued with SNAP-25 (black) or SNAP-23 (blue) and SNAP-25/Syt-7 DKO neurons rescued with SNAP-25 (red), or SNAP-23 (green). B: Expression of SNAP-23 in SNAP-25 KO neurons (blue) led to reduced peak EPSC amplitudes in SNAP-25 KO neurons, which were further significantly reduced in the absence of Syt-7 (green). In contrast, syt-7 deletion (red: SNAP-25 expression in SNAP-25/Syt-7 DKO neuron) was without consequence in the presence of SNAP-25 (black: SNAP-25 expression in SNAP-25 KO neuron). C: SNAP-23 rescued neurons displayed a 3–4 fold reduction in the charge transferred upon a stimulus; this reduction was independent of Syt-7 expression. D: The time-to-peak (EPSC) was prolonged in SNAP-23-rescued SNAP-25 KO neurons. Additional deletion of Syt-7 leads to a total of 50-fold increase in the time-to-peak. E: Spontaneous mEPSC amplitudes were unchanged in all four conditions. F: The frequency of mEPSCs was increased when SNAP-23 was expressed in SNAP-25 KO neurons, and further significantly increased when SNAP-23 was expressed in the SNAP-25/Syt-7 double KO background. G: Explanation of abbreviated labeling, used in Fig. 2–5. * = p<0.05; ** = p<0.01; *** = p<0.001.

Expression of SNAP-23 in SNAP-25 KO glutamatergic neurons created strongly asynchronous EPSCs (Fig. 2A, blue trace; see Fig. 2G for explanation of labeling in this and later figures) with reduced amplitude (Fig. 2B, blue bar) and charge transfer (Fig. 2C) and increased time-to-peak (Fig. 2D), in agreement with previous findings [35]. Expression of SNAP-23 in SNAP-25/Syt-7 DKO cells markedly exacerbated the situation, causing even lower amplitudes (Fig. 2A, B, green traces/bars) and almost 10-fold longer time-to-peak (Fig. 2D), whereas the total charge transfer was only slightly and non-significantly reduced by the elimination of syt-7 (Fig. 2C). In control experiments, we expressed SNAP-25 in SNAP-25 KO and SNAP-25/Syt-7 DKO cells. These experiments showed indistinguishable rescue of synchronized release, as judged by amplitudes, charge and time-to-peak (Fig. 2A–D, black and red traces/bars, respectively). Thus, the lack of syt-7 has no detectable consequences for single EPSCs in the presence of SNAP-25, in agreement with previous findings in cortical cultures [19].

Spontaneous release was assessed in naïve neurons that had not been previously stimulated by high-frequency trains or hypertonic solution (Fig. 2A, E–F). In the presence of tetrodotoxin (TTX), the frequency of spontaneous release events (mEPSCs) was significantly increased after expression of SNAP-23 in SNAP-25 KO neurons, and this increase was further significantly augmented by the absence of syt-7 (from to 11.4±1.0 Hz to 23.1±3.1 Hz, p<0.01) (Fig. 2F). In contrast, the mEPSC frequency was unaffected by syt-7 expression in the presence of SNAP-25 (Fig. 2F). The amplitudes of mEPSCs were unchanged under all conditions (Fig. 2E), which shows that the effect of SNAP-23 and syt-7 is presynaptic in this preparation.

**Figure 3:**
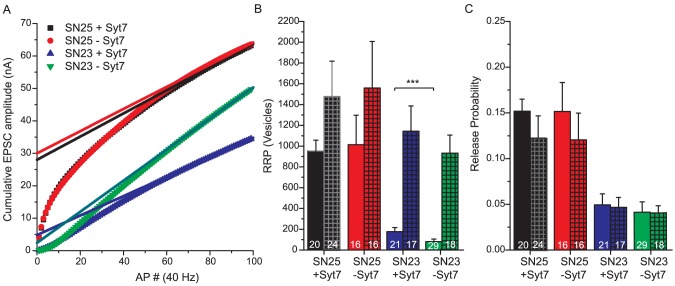
Pool sizes and release probabilities of neurons expression SNAP-25 or SNAP-23 in the presence and absence of synaptotagmin-7. A: Pool sizes estimated by back-extrapolation of cumulative release during a train [41]. The lines are linear fits to the steady state. B: Pool Sizes. Solid Bars: Estimates from train stimulation and back extrapolation Cross-hatched bars: Estimates from sucrose stimulation. The expression of SNAP-23 reduced the pool estimate using back-extrapolation, and syt-7 deletion led to a further decrease. C: Release Probabilities, derived by dividing EPSC amplitudes by the pool size identified by back extrapolation (solid bars), or by dividing the EPSC charge by the charge identified by sucrose application (squared bars). The expression of SNAP-23 reduced the release probability, but it was not further reduced by deleting syt-7.

The releasable vesicle pool (RRP_suc_) size was investigated by application of hypertonic sucrose solution [39], and – in addition – the subpool of vesicles, which is available for fast evoked release (RRP_ev_) was estimated by back-extrapolation of cumulative EPSC amplitudes during a high-frequency train [41] (Fig. 3A). The size of the RRP_suc_ was not significantly different between the four conditions investigated (ANOVA, p>0.85; Fig. 3B, cross-hatched bars), even though there was a tendency towards lower pool sizes in the presence of SNAP-23, which was also noted earlier [35]. The pool sizes determined by back extrapolation of EPSC amplitudes (RRP_ev_) were generally smaller than the ‘sucrose pool’ (Fig. 3B–C, solid bars) [42]. This RRP_ev_ was strongly reduced under SNAP-23 overexpression conditions and further significantly reduced in cells not expressing syt-7 (p<0.001; Fig. 3A–B). This indicates that the fastest releasing part of the RRP is selectively diminished in size in the presence of SNAP-23, such that most vesicles are released ‘reluctantly’ [42], [43]. However, it also has to be considered that the back extrapolation method is less accurate when the release probability is low. Indeed, comparison of the charge evoked by a single action potential with the RRP_suc_ showed that the vesicular release probability, P_vr_, is lower in SNAP-23 expressing cells (Fig. 3C). The same conclusion was reached when the evoked charge was compared to the RRP_ev_ (Fig. 3C) instead of the RRP_suc_.

Overall, our data identify a positive role for syt-7 in evoked release in the presence of SNAP-23, whereas the presence of syt-7 acts to clamp the spontaneous release rate in SNAP-23 expressing neurons.

### Syt-7 suppresses a late phase of release during repetitive stimulation

Asynchronous release components contribute significantly to release during and immediately following high frequency action potential trains [6], [7], [9], [44]. Data from the syt-1 knock-out mouse showed that syt-1 clamps an asynchronous release component driven by one or more alternative calcium sensors, while stimulating synchronous release [45].

**Figure 4:**
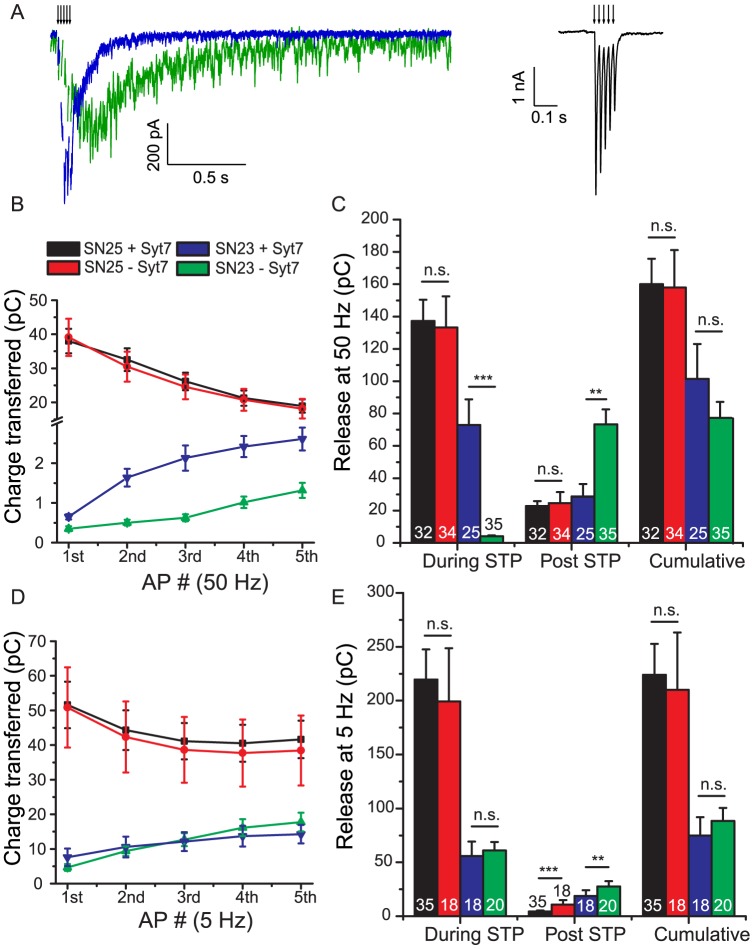
SNAP-23 expressing neurons display asynchronous release, which is shifted to later times by the lack of synaptotagmin-7. A: Example traces (stimulus artifacts and action potential associated currents have been blanked); arrows indicate the five stimuli. Color coding as in Figure 2. B: Charge transfer plotted versus stimuli at 50 Hz. The expression of SNAP-23 reduced the charge, which was further exacerbated by the deletion of syt-7. C: Charge transferred as a result of 50 Hz stimulation (5 stimuli). “During STP”: Integrated release during the interval from the beginning of the train until 20 ms after the train. “Post STP”: Release after the 5th EPSC (+20 ms) until current has relaxed to baseline. “Cumulative”: “During STP”+“Post STP”. Deletion of syt-7 in the presence of SNAP-23 shifted release to after the short train of action potentials. D: As (B), 5 Hz instead of 50 Hz. E: As (C), 5 Hz instead of 50 Hz. *: p<0.05; **: p<0.01; ***:p<0.001.

Brief high-frequency stimulation (5 stimuli @ 50 Hz) in neurons containing SNAP-25 led to short-term synaptic depression of largely synchronized EPSCs (Fig. 4A–B, black and red traces). The majority (86.5%+/−1.3%) of the release took place during the stimulation period (until 20 ms after the last stimulation), and this was not changed in the absence of syt-7 (Fig. 4 B–C, red traces and bars). In the presence of both SNAP-23 and syt-7, the release during the train was strongly decreased, while the release after the train remained unchanged (Fig. 4A–C, blue traces and bars). Interestingly, knock-out of syt-7 in the presence of SNAP-23 shifted almost all release to the period after the train, while the total amount of release remained unchanged (Fig. 4A–C, green traces and bars). Thus, even though release is already asynchronous in the presence of SNAP-23, our data show that syt-7 suppresses an even slower release phase, indicating the existence of biphasic release even on this very slow time scale. The very slow release phase is uncovered at high stimulation frequencies, since at lower frequencies (5 stimuli @ 5 Hz) the post-train release phase was much smaller (Fig. 4D–E). However, even at 5 Hz, the post-train release was significantly higher in the absence of syt-7 (Fig. 4E), regardless of the expression of SNAP-23 or SNAP-25.

**Figure 5:**
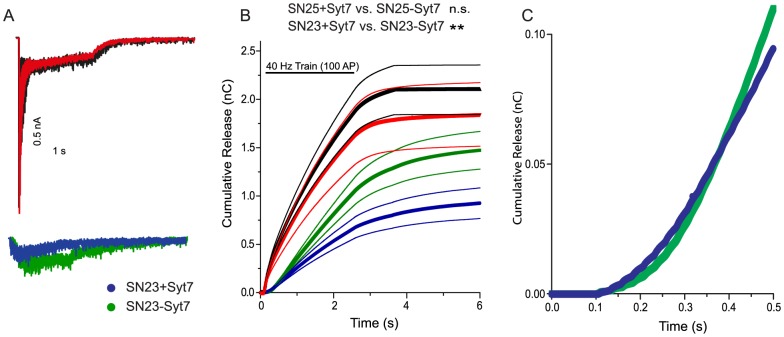
Syt-7 limits the build-up of release during prolonged stimulation in the presence of SNAP-23. A: Examples of 40 Hz, 100 AP trains in the four groups. Color coding as in Figure 2. B: Cumulative release during and after a 40 Hz, 100 AP train. Train stimulation is indicated by a horizontal bar. Shown are SNAP-25 KO neurons rescued with SNAP-25 (black), or SNAP-23 (blue), compared to SNAP-25/Syt-7 DKO neurons rescued with SNAP-25 (red) and SNAP-23 (green). The thick lines are means of all experiments; thin lines indicate the mean ± SEM (number of cells: SNAP-25+syt7, n = 25 cells; SNAP-25−syt7, n = 31 cells; SNAP-23+syt7, n = 19 cells; SNAP-23−syt7, 26 cells). C: Zoom-in of the first 0.5 s of (B), showing that in the presence of SNAP-23, but in the absence of syt-7, release becomes stronger than in the presence of syt-7 at ∼0.4 s (train starts at 0.1 s). **: p<0.01.

Longer train stimulations (100 stimuli @ 40 Hz, Fig. 5) led to build-up of asynchronous release in the presence of SNAP-25 as well as SNAP-23 (Fig. 5A–B). Deletion of syt-7 in SNAP-25 expressing cells mildly decreased the build-up of asynchronous release, in agreement with previous studies (compare red and black curves, Fig. 5B) [21]; however this was not significant when the amount of release after the end of the train was tested with a t-test (Fig. 5B). However, when tested with a Mann-Whitney test, the depression was significant (p = 0.036). It should be noted that in these experiments we relied on the rescue of SNAP-25 KO and SNAP-25/syt-7 DKO neurons with EGFP-SNAP-25, and thus our experimental setting was different from the recent publication [21]. When SNAP-23 was expressed in cells expressing syt-7, the build-up was overall smaller than in the presence of SNAP-25 (Fig. 5B, blue trace). However deleting syt-7 in SNAP-23 expressing cells led to a larger build-up (Fig. 5B), and thus around 250 ms (10 APs) the curves of total released charge crossed, with cells lacking syt-7 having a larger total released charge at later times (Fig. 5C). Overall, repetitive stimulation demonstrate that syt-7 has a role in limiting late occurring asynchronous release in the presence of SNAP-23.

### Vesicles triggered to fuse by SNAP-23 carry syt-1

**Figure 6:**
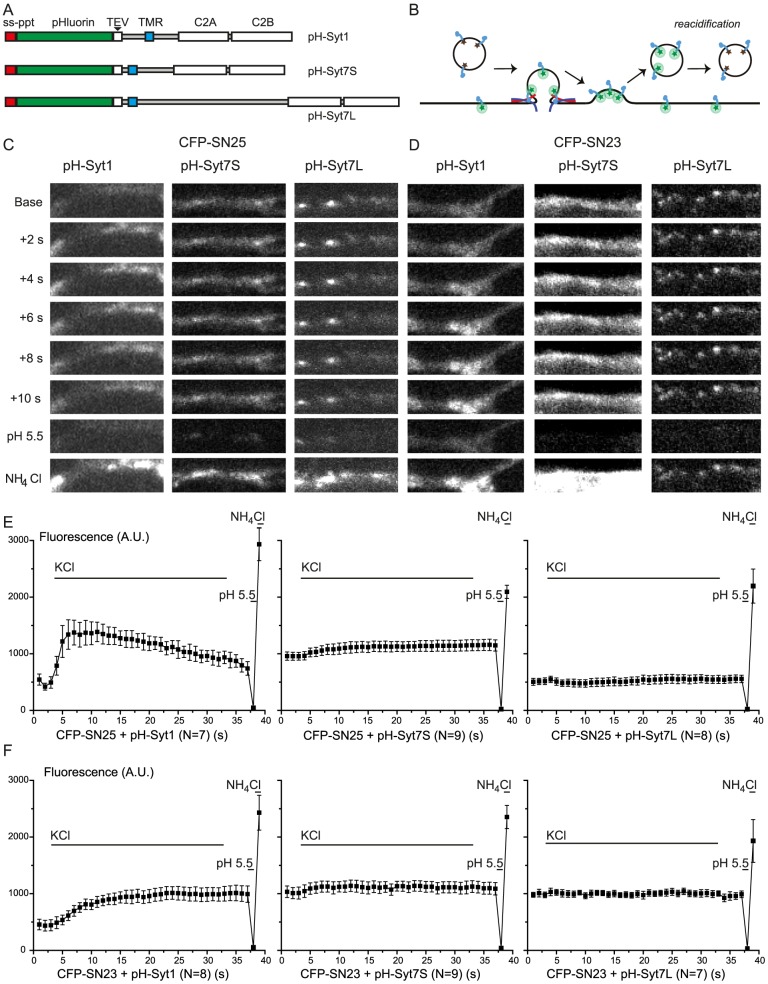
Vesicles fusing asynchronously in the presence of SNAP-23 carry synaptotagmin-1, not synaptotagmin-7. A: pHluorin constructs. pHluorin was fused to the N-terminal of syt-1 or syt-7, preceded by a signal peptide from preprotachykinin (ss-ppt), to ensure proper orientation. For syt-7, two different splice variants were used, a long, and a short. Both have the calcium-binding C2-domains, but they vary in the length of the linker between the Trans Membrane Region (TMR) and the first C2-domain. B. Experimental paradigm. pHluorin-syt situated in acidified intracellular compartments will not be fluorescent, but will gain fluorescence upon fusion with the plasma membrane. C. Example images of pH-Syt1, pH-Syt7S (short isoform), pH-Syt-7L (long isoform) in SNAP-25 KO neurons co-expressing CFP-SNAP-25. At 0 s, the neurons are exposed to high-K solution (45 mM K^+^) to induce vesicular release. “Base” is the normal extracellular solution. D. Same as C, but cells co-express CFP-SNAP-23. E. Quantification of pHluorin fluorescence in SNAP-25 expressing SNAP-25 KO neurons, for all three pHluorin constructs. High-K exposure has been marked “KCl”. The experiment ended with exposure to a solution with pH 5.5, followed by exposure to an ammonium solution to unquench all pHluorins and reveal the total fluorescence. The fluorescence values have been subtracted by the fluorescence in pH 5.5 solution. ‘N’ gives the number of cells (number of synapses analyzed: 414 for CFP-SNAP-25/pH-Syt-1, 591 for CFP-SNAP-25/pH-Syt-7S, 511 for CFP-SNAP-25/pH-Syt-7L). F. Same as E, but neurons co-express EGFP-SNAP-23. ‘N’ gives the number of cells (number of synapses analyzed: 473 for CFP-SNAP-23/pH-Syt-1, 561 for CFP-SNAP-23/pH-Syt-7S, 439 for CFP-SNAP-23/pH-Syt-7L).

Recent studies indicated that asynchronous release might be driven by a specific subpopulation of vesicles carrying VAMP-4 [46], whereas vesicles containing synaptobrevin-2/VAMP-2 fuse synchronously. This implies that loading of different SNAREs onto the synaptic vesicle might predetermine the fusion kinetics. In analogy to this notion, we here asked whether vesicles causing asynchronous release driven by SNAP-23/syt-7, might carry syt-7 instead of syt-1. To this end we expressed a fusion construct between a pH-sensitive variant of GFP – pHluorin [47] – and either syt-1 or syt-7. The linker region of syt-7 is alternatively spliced, giving rise to different syt-7 isoforms [16], [48]. We chose to fuse pHluorin to a short and a long isoform of syt-7, which both contain the calcium-binding C2-domains, but vary in the length of the linker [16](Fig. 6A). In order to allow imaging of pHluorin constructs in cells expressing SNAP-25 or SNAP-23, we exchanged the EGFP tag on SNAP-25/-23 for ECFP (Enhanced Cyan Fluorescent Protein). This allowed visualization of both ECFP (using 475 nm excitation) and pHluorin (using 490 nm excitation, where ECFP has only 2% of its maximal excitation) using the same filter set. We combined expression of syt-1, -7S (short) or -7L (long) with SNAP-25 or SNAP-23 in neurons from SNAP-25 KO mice, creating a total of 6 conditions (Fig. 6).

Application of high-K (45 mM K^+^-containing) solution to cells expressing CFP-SNAP-25 and pHluorin-Syt-1 led to a rapid (within 2 s) increase in pHluorin fluorescence (Fig. 6C), consistent with fast fusion of vesicles with the plasma membrane. Acid wash (pH 5.5) towards the end of the experiment markedly reduced the fluorescence, whereas application of an ammonium chloride solution (pH 7.4), which neutralizes the pH in acidic compartments, led to maximal fluorescence (Fig. 6C). These findings show that the dynamic fluorescence signal originated from pHluorin. Following quantification, the fluorescence signal at low pH was subtracted to isolate the dynamic pHluorin-signal (Fig. 6E). This analysis clearly showed the rapid increase in fluorescence in the presence of both SNAP-25 and syt-1, as expected from the fast synchronized fusion recorded electrophysiologically. In marked contrast, co-expressing pHluorin-syt-7 – either the short or the long isoform – with SNAP-25 did not reveal strong differences in fluorescence upon stimulation with high-K solution (Fig. 6C, E). A small increase in fluorescence in the presence of syt-7S and a small decrease in the presence of syt-7L were observed (Fig. 6E), but these might be caused by small changes in focal level when exchanging the solutions. Nevertheless, these findings do not rule out trafficking of a small fraction of the syt-7 in response to stimulation, but the fraction of trafficked syt-7 must be much smaller compared to syt-1, consistent with a previous report on syt-7 linked pHluorin [49].

Co-expression of pHluorin-syt-1 with CFP-SNAP-23 led to a much slower fluorescence increase upon stimulation by high-K solution (Fig. 6D, F, compare to Fig. 6E, left panel). This is in nice agreement with the electrophysiological findings of strongly asynchronous release in the presence of SNAP-23. However, the fluorescence overall increased ∼2-fold, not much different from the amplitude encountered in the presence of SNAP-25 (Fig. 6E, left panel), indicating substantial trafficking of syt-1 also in this case. In stark contrast, trafficking of syt-7 – short or long isoforms – in the presence of SNAP-23 was again minimal or absent (Fig. 6F, middle and right panel).

Overall, these experiments show that those vesicles that fuse asynchronously in the presence of SNAP-23 carry syt-1, rather than syt-7, arguing that the propensity for SNAP-23 to associate functionally with syt-7 instead of syt-1 is not due to the recruitment of another vesicle population. Since our electrophysiological data showed that syt-7 is involved in SNAP-23 mediated secretion, whereas imaging does not provide evidence for substantial syt-7 trafficking, it appears most likely that syt-7 acts on SNAP-23 from a location on the plasma membrane.

## Discussion

Using double-knockout mice and viral transduction we show that exchanging SNAP-25 for SNAP-23 in glutamatergic hippocampal autaptic neurons renders neurotransmitter release dependent on endogenous syt-7. These findings identify a new calcium-dependent pathway for synaptic vesicle exocytosis, which might be related to the previously identified α-latrotoxin-dependent pathway [50]. In neurons expressing SNAP-23, syt-7 mediates a distinct asynchronous release component and thus constitutes a calcium-sensor for asynchronous neurotransmitter release. The properties of this sensor can be summarized as follows:

1. Syt-7 synchronizes release in the presence of SNAP-23, but release remains much less synchronous than in the presence of SNAP-25/syt-1.
2. Spontaneous release, which is already stimulated by SNAP-23 overexpression, is further disinhibited by removal of syt7, indicating that syt7 acts as a leaky clamp on spontaneous release.
3. Syt-7 plays no decisive role in vesicle priming, as total evoked charge and the size of the ‘sucrose pool’ are largely unaffected by removal of syt-7.
4. Syt-7 determines short-term plasticity in the presence of SNAP-23 during high frequency stimulation and inhibits delayed release.

The notion that different syt isoforms may act as specific release synchronizers for distinct sets of SNAREs is strikingly supported by the observation that the consequences of removing syt-7 in SNAP-23 expressing cells closely match those found in SNAP-25 expressing cells after eliminating syt-1 or syt-2, except for the much slower time scale in the presence of SNAP-23 and syt-7. Indeed, the knock-out of syt-1 or syt-2 has also been shown to desynchronize overall release in all systems investigated [45], [51]–[54], whereas in autaptic neurons the overall evoked charge and the size of the sucrose pool are either slightly reduced or unchanged [55]–[58]. The latter result, however, cannot be generalized to non-autaptic neurons [45], [58]. Likewise, removal of syt-1 or syt-2 cause disinhibition of spontaneous release in most systems [54], [59]–[62], although not in autaptic neurons [58], [62]. Finally, removal of syt-1 also augments delayed release following action potential trains [45].

The similar consequences of removing syt-7 in the presence of SNAP-23 and syt-1 in the presence of SNAP-25 argue that the general mechanism of exocytosis triggering is conserved between different syt/Q_bc_-SNARE pairs. This indicates that syt-7 also acts as a calcium sensor, although in the absence of direct mutation of the calcium-binding sites, we cannot rule out calcium-independent action of syt-7. However, it seems most likely that syt-1 associates with SNAP-25 to form a fast calcium-sensor, and syt-7/SNAP-23 constitutes a calcium-sensor with very similar qualitative properties, but with much slower overall kinetics of action. This finding agrees with the observation of specific pairing between syt-7 and SNAP-23 in an *in vitro* docking assay [34]. The observation of specific functional pairing between syt-7 and SNAP-23 might explain why a previous study failed to restore secretion in syt-1 KO autaptic hippocampal neurons by expressing syt-7 or syt-1/syt-7 chimeric proteins [63].

A previous study indicated that syt-7 located in lysosomes might associate with plasma membrane SNAP-23 [64]. Neuroendocrine cells such as adrenal chromaffin cells, pancreatic α- and β-cells and derived cell lines express primarily SNAP-25, but also smaller amounts of SNAP-23 [65]–[67]. In these cells, syt-7 contributes significantly to overall release [22]–[24], [26], suggesting the functional interplay of endogenous SNAP-23 with syt-7 during endocrine secretion. It was initially reported that some GABAergic neurons express SNAP-23 instead of SNAP-25 [68], [69], but later studies indicated that GABAergic neurotransmission is absent in the SNAP-25 null mouse [35], [70]. However, SNAP-25 is developmentally down-regulated in some subpopulations of neurons [71], which might rely on SNAP-23. Finally, a role for postsynaptic SNAP-23 in NMDA receptor cycling in glutamatergic neurons was recently described [72]. Two recent studies of central neurons suggested that syt-7 acts as an asynchronous calcium sensor in the absence of syt-1, or during high-frequency stimulation [20], or as an upstream calcium-sensor for vesicle replenishment [21]. Interestingly, the phenotype in adrenal chromaffin combines both findings [22], and we recently suggested that one and the same protein might perform both actions [73]. It remains to be seen whether endogenous SNAP-23 might also play a role during these neuronal functions.

How is the specific coupling between syt-7 and SNAP-23, as indicated by this study and the previous in vitro investigation [34], established on the molecular level? This is hard to understand, because the best-studied putative interaction interfaces between syt-1 and SNAP-25 are largely conserved in syt-7 and SNAP-23. This includes the charged stretch between loop 1 and loop 2 in the C2B-domain of syt-1 [74], and the so-called HA-helix at the ‘bottom’ of the C2B-domain [75]. In SNAP-25, three charges around the middle of the complex, which are involved in interacting with syt-1 [31], [33], are conserved in SNAP-23. It is therefore possible that specificity between syts and Q-SNAREs are conferred by other regions of the protein, for instance the 10 amino acid stretch in the C-terminal half of the SNAP-25 linker domain, which we found to be decisive for the different triggering speeds of SNAP-25 and SNAP-23 in adrenal chromaffin cells [76]. Another possibility is that specificity is indirectly accomplished, via matching of kinetic properties between Ca^2+^-sensors and SNAREs. Syt-7, which is the isoform with the slowest calcium unbinding kinetics [77], might be the only syt, which can couple calcium to membrane fusion over the same, slow time scale as SNAP-23 requires to assemble in a SNARE-complex and trigger membrane fusion.

Our experiments with pHluorin-linked syt-1 and-7 showed that vesicles fusing asynchronously in the presence of SNAP-23 carry syt-1, whereas syt-7 barely cycles between intracellular acidic compartments and the plasma membrane. This finding emphasizes the specificity between SNAREs and syts, as even syt-1 carrying vesicles fuse asynchronously under the influence of syt-7 in the presence of SNAP-23. The lack of substantial syt-7 cycling indicates that syt-7 might act from a position on the plasma membrane, even though we notice that roughly half the protein was present in acidified internal organelles, which might represent dense-core vesicles or lysosomes [49]. A function for plasma membrane syt-7 in vesicle recruitment was recently suggested [21], and, indeed, syt-7 was originally suggested to be a plasma membrane calcium-sensor [16].

**Figure 7:**
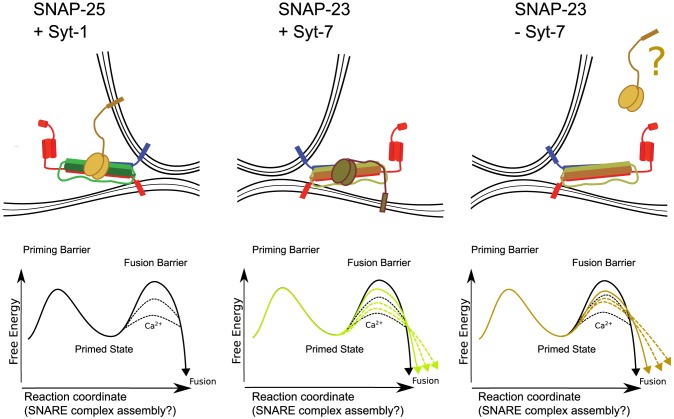
Schematic drawings of possible arrangements of synaptotagmins and Q-SNAREs result in different energy landscapes of fusion. In the control situation, syt-1 and SNAP-25 results in a relatively high energy barrier at rest, and an efficient removal of the fusion barrier under stimulation conditions, leading to fast evoked release and a large difference between evoked and spontaneous fusion rates. In the presence of SNAP-23, syt-7 creates a lower fusion barrier (leading to a higher frequency of spontaneous release than syt-1), which cannot be as efficiently removed, leading to slower (asynchronous) evoked fusion. In the presence of SNAP-23 and in the absence of syt-7 the energy barrier for fusion becomes even more ‘squeezed’ between the calcium-independent and calcium-dependent case, leading to high rates of spontaneous fusion and very asynchronous evoked fusion. The calcium sensor for secretion remains unknown in this situation.

In terms of an energy barrier model for the fusion landscape, our data can be summarized as follows: in the presence of syt-1/SNAP-25, the primed vesicle state is protected from spontaneous fusion by a relatively large energy barrier, which can be rapidly removed to elicit fast fusion (Fig. 7). In the presence of syt-7/SNAP-23, the fusion barrier is not as high, leading to spontaneous release, and fusion is less synchronous because the barrier cannot be removed as efficiently, or as fast. Finally, even in the absence of syt-7 (but in the presence of SNAP-23), there is a – low and thus leaky – fusion barrier, which can be removed slowly to allow very asynchronous fusion (Fig. 7). The identity of the calcium sensor in this case remains unknown. We recently showed that the stability of the N-terminal and the C-terminal part of SNARE-complex play opposing roles for setting the size of the final fusion barrier [78], such that a looser C-terminal end of the complex causes less spontaneous release and a lower release probability, whereas a looser N-terminal end causes more spontaneous release and a slightly higher release probability. Thus, widespread changes in properties of the SNARE-complex and the syt isoforms might underlie these different triggering speeds. In conclusion, our data suggest that cells utilize multiple calcium sensors cooperating with specific Q-SNAREs to elicit membrane fusion via a conserved mechanism – but with widely different kinetics.

## Supporting Information

Data Set S1 The data (single cell values) used to calculate means and SEMs.
(XLSX)
